# Chicken Cholera—Pasture’s New Experiments

**Published:** 1880-06-20

**Authors:** 


### CHICKEN CHOLERA—PASTEUR'S NEW EXPERIMENTS.

Mr. Pasteur's last communication to the Academy of Sciences and the Academy of Medicine upon chicken cholera, is not only the great scientific event of the month, but without doubt contains one of the greatest discoveries of the times. It is still the process of cultivating microscopic beings which forms the basis of the new experiments and which has permitted the grouping together in one sheaf of new facts that give us a glimpse of more correct scientific conceptions of the nature of diseases for the future, of a better treatment and protection, and a prophylaxis such as we have never been able even to dream of.

Under the name of chicken cholera is designated an epidemic disease of very rapid progress which depopulates the poultry yards. Loss of strength, depression, and invincible somnolence characterizes it. A microscopic organism is the cause of this disease. It was discovered by Moritz and has been described particularly by Peroncito and Toussaint, of Toulouse. Of extreme smallness, when it multiplies it forms, with marvelous rapidity, immense quantities of new organisms so minute that they are not measurable by the microscope.

Mr. Pasteur has studied this organism as he has that of the bacteria of malignant pustule (charbon), and has ascertained that it must have a peculiar medium of cultivation; chicken soup (bouillon de poule); other solutions, suitable for the culture of other microscopic organisms (microbes) are absolutely unsuitable to its culture, it is reproduced badly therein, and perishes rapidly. There is a very curious relation between the medium of cultivation and the living individuals, of which one is suited to the development of an infectious disease, while another animal species is absolutely refractory to it.

When this cultivated microbe is inoculated, or when the blood of a fowl dead from cholera is inoculated, the same effects, the same development of disease and rapid death follow, almost certainly, in every case.

Chicken cholera, like the most of the infectious diseases, appears to guarantee the individual that has had it against recurrence. If a fowl has recovered from cholera it may be inoculated without result with the blood of a choleraic fowl, it resists the development of the disease.

Now, and this is the capital point in M. Pasteur's communication, by a peculiar mode of cultivating the microbe he has succeeded in obtaining an enfeebled microbe, which is incapable of killing the fowl but of protecting it against the cholera contagion. The fowl inoculated with the modified microbe is sick, but it does not die, it always survives. After it has regained its health, if it be inoculated with the strong, normal virus, with the blood of a fowl that has died of unmodified chicken cholera, it does not die, it is not even made sick.

Thus, in every respect, M. Pasteur has created vaccination against chicken cholera. He has not yet disclosed his method of thus enfeebling the power of the microbe, but will make it known in an early publication. From this may we not see for the future a limitless field for therapeutics and prophylaxis?

What an influence is exerted by the soil upon the development of the micro-organism has never before been so well demonstrated. Certain animal species are entirely unaffected by it; upon others, the rabbit and the Guinea pig, a local lesion is produced in which the microbe multiplies and preserves its virulence, but does not generalize its action.

Finally, the micro-organism does not develop again in the individual in which it has once developed. Again, the chicken soup in which the microbe has been reproduced in abundance ceases to be a good soil for it. If it is filtered and thus cleared of these organisms which have been abundantly produced, but forming only an insignificant mass, the soup, although suitable for the development of other species of vibrios, no longer permits the production of the cholera microbe; it is worn out for this species as is the organism which has once passed through the disease.

The transmission of this disease from one fowl to another is easy of accomplishment, for nothing more is necessary than to cause a fowl to injest some of the parasites, and it soon falls a prey to the affection. Should the pus from an abscess, caused by inoculating an animal like the Guinea pig with the disease, should some of this pus containing quantities of vibrios be mixed with the food given to fowls, the latter are quickly affected with cholera. Hence there are two methods of contagion: the direct and the indirect which throws light on many facts hitherto obscure.

As to inoculation, there is ordinarily produced a grave local disorder: induration, purulent infiltration and gangrene. The region swarms with micro-organisms. If the fowl has already been vaccinated, the local lesion is insignificant, the point affected throws off a small slough of no consequence.

Such, in short, are the facts which M. Pasteur has just shown to the

Academy when he reminded the members of the works of Davaine, Chauveau, Klebs, Koch as well as his own, upon the part taken by vibrios in virulent diseases and upon their transformations. By experiment he boldly attacks the most difficult problems in general pathology, and we already see him in possession of most important information concerning the modes of transmission of diseases and the power of resistance acquired by the organism which has withstood one attack.

It is to be seen that what is called the germ theory has entered upon a new phase. Pasteur's communication has produced a considerable sensation in the learned world, and we are willing to leave this sketch in the hands of the reader without further comment.—*Journal de Medecine et de Chirurgie pratiques.*

